# Oral amoxicillin-clavulanate: an alternative treatment to refractory *Nocardia* keratitis—a case report

**DOI:** 10.1128/asmcr.00223-25

**Published:** 2026-04-23

**Authors:** Prathishtha Pitchyaiah, Rachel A. F. Wozniak

**Affiliations:** 1 Department of Ophthalmology, University of Rochester School of Medicine and Dentistry 12299, Rochester, New York, USA; Rush University Medical Center, Chicago, Illinois, USA

**Keywords:** systemic amoxicillin-clavulanate, antibiotic resistance, *Nocardia amikacinitolerans* keratitis

## Abstract

**Background:**

Bacterial keratitis (bacterial corneal infection) is a serious, vision-threatening disease caused by a wide range of bacterial species. The gram-positive bacterium *Nocardia* spp. is increasingly recognized in keratitis, yet is particularly challenging to treat due to delayed diagnosis and limited efficacy of current ophthalmic therapeutics. Antimicrobial resistance among clinical *Nocardia* isolates is also rising, further complicating patient outcomes. Herein, we report a case of *Nocardia amikacinitolerans* keratitis, resistant to amikacin, ciprofloxacin, moxifloxacin, clarithromycin, and ceftriaxone, resulting in a complicated therapeutic course.

**Case Summary:**

A 53-year-old female presented with bacterial keratitis of the right eye, which over the course of 32 months progressed to scleritis (infection of the sclera) and a progressive, significant decrease in visual acuity to hand motion despite exhaustive topical and systemic antimicrobial therapies. While there is a general lack of efficacy of systemic therapies for bacterial keratitis, oral amoxicillin-clavulanate ultimately proved successful in resolving the infection.

**Conclusion:**

As *Nocardia* spp. ocular infections become more prevalent, this report underscores the highly virulent nature of this pathogen and provides insights into potential therapeutic modalities in the setting of multidrug resistance.

## INTRODUCTION

Bacterial keratitis, or bacterial infection of the cornea, causes at least 2 million new cases of blindness annually (1). While a diversity of bacterial species can cause corneal infections, clinical presentations can be strikingly similar and include corneal ulceration, edema, and immune infiltration, making microbial cultures essential to guide therapy (2). *Nocardia spp*. are a gram-positive, weakly acid-fast, aerobic bacteria that are increasingly identified in bacterial keratitis, particularly in Southeast Asia, and may account for upwards of 1.7%–8.3% of bacterial keratitis cases worldwide (3–11). Clinically, *Nocardia* keratitis has been described as an indolent infection with multifocal, satellite corneal stromal immune infiltrates in a “wreath-like” pattern (12, 13). Risk factors for *Nocardia* keratitis overlap with other bacterial corneal infections and include prior ocular surgery, immunosuppression, and contact lens wear, but more specifically, ocular trauma with organic material (13, 14). While the standard of care for bacterial keratitis commonly includes a combination of topical fluoroquinolones, vancomycin, ceftazidime, or tobramycin, topical amikacin is currently considered a first-line therapeutic for *Nocardia* keratitis per the American Academy of Ophthalmology Preferred Practice Guidelines (15, 16). As a second-line therapy, oral trimethoprim-sulfamethoxazole (TMP-SMX) has been used with success in several case reports from the US (6), India (17, 18), Sweden (8), and Spain (7).

This report presents a case of infectious keratitis caused by the less common, drug-resistant species *Nocardia amikacinitolerans* that progressed to infectious scleritis. The prolonged therapeutic course highlights the clinical challenges in the management of *Nocardia* keratitis, particularly in the setting of antibiotic resistance, and describes clinical success with oral amoxicillin-clavulanate, a therapy that, to our knowledge, has not been previously used for this indication.

## CASE PRESENTATION

A 53-year-old female presented to an academic medical center with redness and a foreign body sensation in her right eye for 3 weeks. The patient was immunocompetent and denied any history of trauma, contact lens use, recent hot tub use, cold sores, or recent travel.

**Fig 1:**
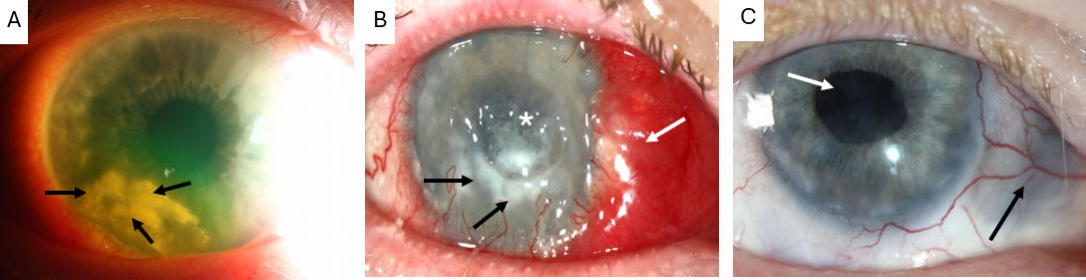
Slit lamp photographs of the right eye. (**A**) Ten days after presentation with retro-illumination and fluorescein dye indicating areas of multifocal mid-stromal infiltrates (black arrows). (**B**) Seven months after presentation indicating multifocal mid-stromal infiltrates (black arrows), inferior corneal neovascularization, central corneal thinning (asterisk), and severe scleritis nasally (white arrow), with overlying conjunctival chemosis and severe injection. (**C**) Finally, 2.5 years after presentation, scleral thinning (black arrow) and diffusely patchy corneal scarring (white arrow).

On exam, the patient’s best-corrected visual acuity was 20/25 in the right eye and 20/20 in the left eye, with normal intraocular pressures and reactive pupils. Slit lamp examination of the right eye was notable for scleral injection and a peripheral corneal epithelial defect measuring 4.5 mm × 2.5 mm with adjacent multifocal stromal infiltrates. A corneal swab using a standard E-swab (19) was obtained for Gram stain, aerobic, anaerobic, and fungal cultures, and PCR detection of herpes simplex virus and varicella zoster virus. The initial Gram stain was negative (no organisms seen); thus, while awaiting definitive culture results, empiric, broad-spectrum therapy was initiated, which included hourly topical vancomycin 25 mg/mL and tobramycin 14 mg/mL, as well as topical prednisolone acetate 1% twice daily and oral valacyclovir 500 mg twice daily. The University of Rochester clinical microbiology laboratory inoculated the E-swab on blood and chocolate agar plates as well as in thioglycolate broth. While broth cultures were negative, several dry, white-appearing colonies grew on both agar plates. A single colony from the blood agar plate was sent for MALDI-TOF analysis using the Bruker MALDI-TOF MS system and resulted in unvalidated *Nocardia*. On day 7, a confirmatory Gram stain was repeated, which revealed bacilli with a branching and beaded appearance consistent with *Nocardia*, and a modified Kinyoun acid-fast stain was performed, which was positive. Based on these results and due to worsening corneal stromal infiltrates (Fig. 1A), topical amikacin (20 mg/mL) every 2 h was initiated.

On day 13, 16S rRNA sequencing was completed using a single colony and revealed *Nocardia amikacinitolerans*. Antimicrobial susceptibility testing was performed utilizing a Sensititre gram-positive plate. Results indicated resistance to amikacin but susceptibility to amoxicillin-clavulanate, imipenem, linezolid, tobramycin, and trimethoprim-sulfamethoxazole (TMP-SMX) (Table 1). Given the continued lack of clinical improvement, a 2-week course of oral TMP-SMX 160–800 mg twice daily was prescribed.

**TABLE 1:** Susceptibility profiles of *Nocardia amikacinitolerans* cultures taken at initial presentation and at 2 months^*a*^

Antibiotic	*N. amikacinitolerans* Initial presentation		*N. amikacinitolerans* 2 months	
	MIC (mcg/mL)	Susceptibility	MIC (mcg/mL)	Susceptibility
**Amikacin**	**≥64.0**	**Resistant**	**≥64.0**	**Resistant**
**Amoxicillin-clavulanic acid**	**4.0/2.0**	**Susceptible**	**4.0/2.0**	**Susceptible**
Ceftriaxone	≥128	Resistant	4.0	Susceptible
Ciprofloxacin	16.0	Resistant	16.0	Resistant
Clarithromycin	≥32	Resistant	≥32	Resistant
**Doxycycline**	**2.0**	**Intermediate**	**1.0**	**Susceptible**
Imipenem	4.0	Susceptible	4.0	Susceptible
Linezolid	2.0	Susceptible	2.0	Susceptible
Minocycline	2.0	Susceptible	0.5	Susceptible
**Moxifloxacin**	**8.0**	**Resistant**	**8.0**	**Resistant**
**Tobramycin**	**≤2.0**	**Susceptible**	**≤2.0**	**Susceptible**
**Trimethoprim-sulfamethoxazole (TMP-SMX**)	**0.25/4.75**	**Susceptible**	**0.5/9.5**	**Susceptible**

^*a*^ Antimicrobial susceptibility testing was performed in-house using a Sensititre gram-positive plate. Antibiotics used in this case are bolded.

However, over the subsequent two months, there was progressive worsening of clinical signs, symptoms, and visual acuity. During this time, the patient was treated with a variety of topical therapies including vancomycin (25 mg/mL) every 2 h, tobramycin (14 mg/mL) every 2 h, cyclopentolate (1%) 3 times daily, prednisolone acetate (1%) daily, moxifloxacin (0.5%) 4 times daily, polymyxin B/trimethoprim (10,000 units/ 1 mg/mL) 4 times daily, and tobramycin/dexamethasone (0.3–0.1%) ointment 3 times daily. After 2 months, due to minimal clinical improvement, a second corneal culture was obtained and again speciated as *N. amikacinitolerans*. Antimicrobial susceptibility testing was repeated and revealed resistance to amikacin, ciprofloxacin, moxifloxacin, and clarithromycin (Table 1).

**Fig 2:**
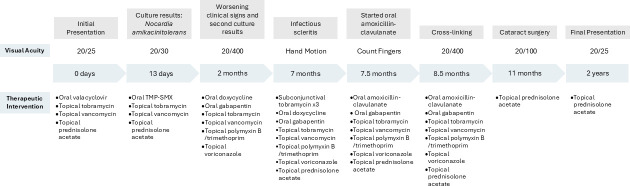
Timeline of visual acuity progression, key results, procedures, and treatment course. Anti-infectives included valacyclovir, tobramycin, vancomycin, polymyxin B/trimethoprim, voriconazole, and amoxicillin-clavulanate. Prednisolone acetate was used for ocular inflammation, gabapentin for pain management, and doxycycline was used to mitigate corneal melting.

Following these results, the patient was treated with an increased dose of topical polymyxin B/trimethoprim (10,000 units/1 mg/mL) every 2 h, tobramycin (14 mg/mL) every 2 h, and oral doxycycline 100 mg daily. Over the next 3 months, the patient’s pain slowly improved and clinical findings (corneal edema, stromal infiltrates) stabilized. However, 4 months after the second culture (7 months after initial presentation), the patient developed an inflammatory scleral nodule associated with severe scleral injection and edema, consistent with infectious scleritis, as well as central corneal thinning (Fig. 1B). Given the reported susceptibility to tobramycin and clinical precedence (20), subconjunctival tobramycin (0.2 cc of 14 mg/mL) was injected into the subconjunctival space above the scleral nodule three times. Unfortunately, no clinical improvement was seen, and visual acuity decreased to hand motion. Despite a lack of published evidence in the ophthalmology literature, a course of oral amoxicillin-clavulanate (875–125 mg) twice daily was initiated. Within 11 days, the patient reported complete resolution of ocular pain, and her exam was notable for a significant decrease in scleritis as indicated by decreased scleral injection and resolution of the inflammatory nodule, stromal infiltrates, and corneal edema. The patient completed a 5-week course of twice-daily oral amoxicillin-clavulanate (875–125 mg). Of note, 4 weeks into the amoxicillin-clavulanate regimen, UV corneal cross-linking was also performed to strengthen the cornea due to existing corneal thinning. Over the following 6 months, the patient was tapered off all antibiotics, and her visual acuity improved from hand motion to 20/100. She underwent cataract surgery and ultimately achieved a best-corrected visual acuity of 20/25 using a gas permeable contact lens. Her final exam was notable for scleral thinning around the prior inflammatory nodule, diffusely patchy corneal scarring, and mild corneal thinning (Fig. 1C). The timeline of disease progression, treatment, and key points is shown in Fig. 2.

## DISCUSSION

Currently, topical amikacin, an aminoglycoside, is considered a first-line treatment for *Nocardia* keratitis (3). However, the emergence of amikacin-resistant strains, in particular *N. amikacinitolerans,* has prompted alternative approaches to effectively manage these infections, including oral TMP-SMX (3, 8, 21, 22).

The incidence of *N. amikacinitolerans* keratitis is not well known, particularly in the United States, where *Nocardia* keratitis remains a rare cause of corneal infections. The only other published report of *N. amikacinitolerans* keratitis is from Sweden in 2016, in which a patient was successfully treated with oral TMP-SMX after failure of conventional topical regimens, including amikacin (8). More recently, a 2022 study reported the susceptibility profiles of 26 *Nocardia* spp. corneal isolates collected over a 7-year period in Florida, of which 11 were *N. amikacinitolerans*. This *in vitro* study revealed amikacin resistance in 64% of isolates, including all 11 *N. amikacinitolerans* strains tested. While the therapeutic regimens or clinical outcomes associated with these isolates are not known, susceptibility testing led the authors to recommend oral TMP-SMX as the preferred antibiotic agent in treating amikacin-resistant *Nocardia* keratitis (21). While it is generally accepted that topical administration of antimicrobials will achieve drug concentrations well above the susceptibility breakpoints, thereby potentially overcoming resistance, in the present case, lack of clinical improvement with amikacin prompted initiation of systemic TMP-SMX.

In *Nocardia* keratitis cases in which topical amikacin and oral TMP-SMX are clinically ineffective, there are no known published alternatives. In extra-ocular nocardiosis, while TMP-SMX is the most well-studied and utilized treatment, amoxicillin-clavulanate and linezolid have also been used successfully (23–32). However, it is important to note that ophthalmic efficacy of systemic antimicrobial therapeutics is inconsistent, secondary to variable drug concentrations that can be achieved in ocular tissues, particularly the cornea. To our knowledge, this is the first reported case of *Nocardia* keratitis responding to oral amoxicillin-clavulanate, suggesting that this drug may penetrate the cornea adequately and serve as an adjunctive therapy in ocular *Nocardia* infections.

Finally, it should be acknowledged that the patient was on topical steroids during her treatment course. Importantly, the Steroids for Corneal Ulcer Trial demonstrated that in bacterial keratitis, topical steroids did not delay healing or impact visual outcomes compared to placebo at 3 months (33). Of note, in a subsequent subgroup analysis, in *Nocardia* keratitis, while topical steroid use resulted in slightly larger corneal scarring (<1 mm) at 12 months vs placebo, there was no statistical difference in visual outcomes (34). In the current case, the benefit of steroids in the setting of an inflammatory scleral nodule and corneal neovascularization far outweighed the risk of increased corneal scarring and was unlikely to have contributed to her protracted clinical course.

In summary, *Nocardia* keratitis remains a difficult-to-manage infection that can significantly damage ocular tissues (12, 35). While topical amikacin and oral TMP-SMX have been used previously with success, this case demonstrates that amoxicillin-clavulanate may represent a viable alternative in select cases.

## References

[1] Whitcher JP, Srinivasan M, Upadhyay MP. 2001. Corneal blindness: a global perspective. Bull World Health Organ 79:214–221. doi:10.1590/S0042-96862001000300009 11285665 PMC2566379

[2] Al-Mujaini A, Al-Kharusi N, Thakral A, Wali UK. 2009. Bacterial keratitis: perspective on epidemiology, clinico-pathogenesis, diagnosis and treatment. Sultan Qaboos Univ Med J 9:184–195. doi:10.18295/2075-0528.2789 21509299 PMC3074777

[3] Lalitha P. 2009. Nocardia keratitis. Curr Opin Ophthalmol 20:318–323. doi:10.1097/ICU.0b013e32832c3bcc 19387343

[4] Garg P, Rao GN. 1999. Corneal ulcer: diagnosis and management. Community Eye Health 12:21–23. 17491983 PMC1706003

[5] Troumani Y, Touhami S, Beral L, David T. 2015. Corneal nocardiosis mistaken for fungal infection. J Fr Ophtalmol 38:e7–e9. doi:10.1016/j.jfo.2014.05.011 25455558

[6] Patel R, Sise A, Al-Mohtaseb Z, Garcia N, Aziz H, Amescua G, Pantanelli SM. 2015. Nocardia asteroides keratitis resistant to amikacin. Cornea 34:1617–1619. doi:10.1097/ICO.0000000000000634 26418432

[7] Prieto-Borja L, García-Coca M, Ustratova I, Alejandre Alba N. 2017. Keratitis due to Nocardia nova after cataract surgery. Enferm Infecc Microbiol Clin 35:57–58. doi:10.1016/j.eimc.2016.06.004 27448807

[8] Johansson B, Fagerholm P, Petranyi G, Claesson Armitage M, Lagali N. 2017. Diagnostic and therapeutic challenges in a case of amikacin‐resistant Nocardia keratitis. Acta Ophthalmol (Copenh) 95:103–105. doi:10.1111/aos.13182 27572657

[9] Shah P, Zhu D, Culbertson WW. 2017. Therapeutic femtosecond laser-assisted lamellar keratectomy for multidrug-resistant Nocardia Keratitis. Cornea 36:1429–1431. doi:10.1097/ICO.0000000000001318 28834821

[10] Gieger A, Waller S, Pasternak J. 2017. Nocardia Arthritidis Keratitis: case report and review of the literature. Nep J Oph 9:91–94. doi:10.3126/nepjoph.v9i1.17543 29022964

[11] Mosenia A, Nguyen AH, Mandel MR, Seitzman GD. 2022. Nocardia sienata: a new causative species of infectious keratitis. BMJ Case Rep 15:e247850. doi:10.1136/bcr-2021-247850 PMC8961103 35338040

[12] DeCroos FC, Garg P, Reddy AK, Sharma A, Krishnaiah S, Mungale M, Mruthyunjaya P, Hyderabad Endophthalmitis Research Group. 2011. Optimizing diagnosis and management of nocardia keratitis, scleritis, and endophthalmitis: 11-year microbial and clinical overview. Ophthalmology 118:1193–1200. doi:10.1016/j.ophtha.2010.10.037 21276615

[13] Sridhar MS, Gopinathan U, Garg P, Sharma S, Rao GN. 2001. Ocular nocardia infections with special emphasis on the cornea. Surv Ophthalmol 45:361–378. doi:10.1016/s0039-6257(00)00207-1 11274691

[14] Bharathi MJ, Ramakrishnan R, Vasu S, Chirayath A, Palaniappan R, Meenakshi. 2003. Nocardia asteroides keratitis in South India. Indian J Med Microbiol 21:31–36. doi:10.1016/S0255-0857(21)03006-1 17642971

[15] Gurnani B, Kaur K. 2025. Bacterial Keratitis. *In* StatPearls. StatPearls Publishing. 34662023

[16] Rhee MK, Ahmad S, Amescua G, Cheung AY, Choi DS, Jhanji V, Lin A, Mian SI, Viriya ET, Mah FS, Varu DM, American Academy of Ophthalmology Preferred Practice Pattern Cornea/External Disease Panel. 2024. Bacterial Keratitis preferred practice pattern. Ophthalmology 131:87–P133. doi:10.1016/j.ophtha.2023.12.03537598860

[17] Alhemyari MH, Satarasi P, Joseph J, Bagga B. 2024. Nocardia keratitis after small incision lenticule extraction (SMILE). BMJ Case Rep 17:e259486. doi:10.1136/bcr-2023-259486 38749526

[18] Bellala MM, Tandra PS, Bagga B, Madduri B. 2023. Nocardia keratitis presenting as an anterior chamber ball of exudates and its management. BMJ Case Rep 16:e251647. doi:10.1136/bcr-2022-251647 PMC9945109 36810335

[19] Van Horn KG, Audette CD, Sebeck D, Tucker KA. 2008. Comparison of the Copan ESwab system with two Amies agar swab transport systems for maintenance of microorganism viability. J Clin Microbiol 46:1655–1658. doi:10.1128/JCM.02047-07 18353935 PMC2395074

[20] Cunha LP da, Juncal V, Carvalhaes CG, Leão SC, Chimara E, Freitas D. 2018. Nocardial scleritis: a case report and a suggested algorithm for disease management based on a literature review. Am J Ophthalmol Case Rep 10:1–5. doi:10.1016/j.ajoc.2018.01.018 29780901 PMC5956651

[21] Adre E, Maestre-Mesa J, Durkee H, Arboleda A, Flynn H Jr, Amescua G, Parel J-M, Miller D. 2022. Nocardia keratitis: amikacin nonsusceptibility, risk factors, and treatment outcomes. J Ophthalmic Inflamm Infect 12:11. doi:10.1186/s12348-022-00287-1 35247126 PMC8898206

[22] Soleimani M, Masoumi A, Khodavaisy S, Heidari M, Haydar AA, Izadi A. 2020. Current diagnostic tools and management modalities of Nocardia keratitis. J Ophthalmic Inflamm Infect 10:36. doi:10.1186/s12348-020-00228-w 33263838 PMC7710777

[23] Smego RA, Moeller MB, Gallis HA. 1983. Trimethoprim-sulfamethoxazole therapy for Nocardia infections. Arch Intern Med 143:711–718. 6340623

[24] Margalit I, Lebeaux D, Tishler O, Goldberg E, Bishara J, Yahav D, Coussement J. 2021. How do I manage nocardiosis? Clin Microbiol Infect 27:550–558. doi:10.1016/j.cmi.2020.12.019 33418019

[25] Lebeaux D, Bergeron E, Berthet J, Djadi-Prat J, Mouniée D, Boiron P, Lortholary O, Rodriguez-Nava V. 2019. Antibiotic susceptibility testing and species identification of Nocardia isolates: a retrospective analysis of data from a French expert laboratory, 2010-2015. Clin Microbiol Infect 25:489–495. doi:10.1016/j.cmi.2018.06.013 29933049

[26] Steingrube VA, Wallace RJ Jr, Brown BA, Pang Y, Zeluff B, Steele LC, Zhang Y. 1991. Acquired resistance of Nocardia brasiliensis to clavulanic acid related to a change in beta-lactamase following therapy with amoxicillin-clavulanic acid. Antimicrob Agents Chemother 35:524–528. doi:10.1128/AAC.35.3.524 2039203 PMC245043

[27] Wang H, Zhu Y, Cui Q, Wu W, Li G, Chen D, Xiang L, Qu J, Shi D, Lu B. 2022. Epidemiology and antimicrobial resistance profiles of the Nocardia species in China, 2009 to 2021. Edited by B. C. Prokesch. Microbiol Spectr 10:e01560-21. doi:10.1128/spectrum.01560-21 35234511 PMC9045199

[28] Yetmar ZA, Marty PK, Clement J, Miranda C, Wengenack NL, Beam E. 2025. State-of-the-art review: modern approach to nocardiosis-diagnosis, management, and uncertainties. Clin Infect Dis 80:e53–e64. doi:10.1093/cid/ciae643 40305688

[29] Bonifaz A, Flores P, Saúl A, Carrasco-Gerard E, Ponce RM. 2007. Treatment of actinomycetoma due to Nocardia spp. with amoxicillin-clavulanate. Br J Dermatol 156:308–311. doi:10.1111/j.1365-2133.2006.07557.x 17223871

[30] Ji Y, Su F, Hong X, Chen M, Zhu Y, Cheng D, Ge Y. 2023. Successful treatment with amoxicillin-clavulanic acid: cutaneous nocardiosis caused by Nocardia brasiliensis. J Dermatolog Treat 34:2229467. doi:10.1080/09546634.2023.2229467 37394975

[31] Aguilar-Molina H, Toussaint-Caire S, Arenas R, Xicohtencatl-Cortes J, Martínez-Chavarría LC, Hernández-Castro R, Rodriguez-Cerdeira C. 2025. Primary cutaneous nocardiosis (Lymphangitic Type) in an immunocompetent patient: a case report. Microorganisms 13:1022. doi:10.3390/microorganisms13051022 40431195 PMC12114168

[32] Hidayah RMN, Gunawan H, Dwiyana RF, Anandita R, Fauziah N, Usman HA, Annadya S. 2025. A rare case of actinomycetoma in pregnancy: effective treatment with the combination of amoxicillin-clavulanic acid. Int J Womens Health 17:2493–2498. doi:10.2147/IJWH.S527336 40800062 PMC12341821

[33] Srinivasan M, Mascarenhas J, Rajaraman R, Ravindran M, Lalitha P, Glidden DV, Ray KJ, Hong KC, Oldenburg CE, Lee SM, Zegans ME, McLeod SD, Lietman TM, Acharya NR, Steroids for Corneal Ulcers Trial Group. 2012. The steroids for corneal ulcers trial: study design and baseline characteristics. Arch Ophthalmol 130:151–157. doi:10.1001/archophthalmol.2011.303 21987581 PMC3830555

[34] Srinivasan M, Mascarenhas J, Rajaraman R, Ravindran M, Lalitha P, O’Brien KS, Glidden DV, Ray KJ, Oldenburg CE, Zegans ME, Whitcher JP, McLeod SD, Porco TC, Lietman TM, Acharya NR, Steroids for Corneal Ulcers Trial Group. 2014. The steroids for corneal ulcers trial (SCUT): secondary 12-month clinical outcomes of a randomized controlled trial. Am J Ophthalmol 157:327–333. doi:10.1016/j.ajo.2013.09.025 24315294 PMC3946996

[35] Sridhar MS, Cohen EJ, Rapuano CJ, Lister MA, Laibson PR. 2002. Nocardia asteroides sclerokeratitis in a contact lens wearer. CLAO J Off Publ Contact Lens Assoc Ophthalmol Inc 28:66–68. 12054371

